# Neuroprotective effects of sesamol against LPS-induced spatial learning and memory deficits are mediated via anti-inflammatory and antioxidant activities in the rat brain

**DOI:** 10.22038/AJP.2022.21403

**Published:** 2023

**Authors:** Zahra Beheshtimanesh, Ziba Rajaei

**Affiliations:** *Department of Physiology, School of Medicine, Isfahan University of Medical Sciences, Isfahan, Iran*

**Keywords:** Sesamol, Lipopolysaccharide, Cytokine, Memory, Oxidative stress, Neuroinflammation, Rat

## Abstract

**Objective:**

Sesamol is a phenolic lignan extracted from sesame seeds, and it possesses anti-inflammatory and antioxidant activities. Lipopolysaccharide (LPS) is known to produce neuroinflammatory responses and memory impairment. The current study aimed to investigate the protective influence of sesamol against LPS-mediated neuroinflammation and memory impairment.

**Materials and Methods:**

Sesamol (10 and 50 mg/kg) was injected to Wistar rats for two weeks. Then, animals received LPS injection (1 mg/kg) for five days, while treatment with sesamol was performed 30 min before LPS injection. Spatial learning and memory were assessed by the Morris water maze (MWM), two hours after LPS injection on days 15-19. Biochemical assessments were performed after the end of behavioral experiments.

**Results:**

LPS-administered rats showed spatial learning and memory deficits, since they spent more time in the MWM to find the hidden platform and less time in the target quadrant. Besides these behavioral changes, tumor necrosis factor-α (TNF-*α*) and lipid peroxidation levels were increased, while total thiol level was decreased in the hippocampus and/or cerebral cortex. In addition, sesamol treatment (50 mg/kg) for three weeks decreased the escape latency and increased the time on probe trial. Sesamol also reduced lipid peroxidation and TNF-α level, while enhanced total thiol level in the brain of LPS-exposed rats.

**Conclusion:**

Supplementation of sesamol attenuated learning and memory impairments in LPS-treated rats via antioxidative and anti-inflammatory activities in the rat brain.

## Introduction

Alzheimer’s disease (AD) is the most prevalent type of dementia that devotes up to seventy percent of dementia cases (WHO, 2021). AD is diagnosed by cognitive and memory impairments, amyloid plaques and tau tangles in the hippocampus, entorhinal cortex, amygdala and basal forebrain (Brown, 2019; Perl, 2010). Neuroinflammation is a crucial factor involved in the occurrence and progression of AD (Rather et al., 2021). Prior studies have reported the existence of astrocytes and microglia around the amyloid plaques in AD (Varnum and Ikezu, 2012), as well as increased expression of different proinflammatory cytokines, including tumor necrosis factor-α (TNF-*α*), interleukin-1β (IL-1β) and free radicals in the blood samples and brain of AD patients (Akiyama et al., 2000; Ganguly et al., 2021).

Lipopolysaccharide (LPS) is a bacterial endotoxin which acts as an inducer of inflammation and participates in neuroinflammation and eventually neurodegeneration (Batista et al., 2019). Systemic LPS injection leads to inflammatory responses by binding to toll-like receptor-4 (TLR4) and subsequently NF-κB transcriptional activation of different proinflammatory genes such as TNFα, IL-1β and IL-6 (Bryant et al., 2010; Morris et al., 2015). This event impairs neuronal function in the hippocampus and eventually leads to neuronal death and memory dysfunction (Zakaria et al., 2017). LPS also produces high amounts of reactive oxygen species (ROS), which ultimately causes neuronal death and memory dysfunction (Khan et al., 2016; Amooheydari et al., 2022). Elevated ROS formation leads to oxidative damage to proteins, lipids and nucleic acids, resulting in deregulation of cellular function and neurodegeneration (Ammari et al., 2018). LPS also enhances Aβ formation and aggregation (Zhu et al., 2021) and tau hyperphosphorylation (Gardner et al., 2016).

Sesamol (3, 4-methylenedioxyphenol) is a phenolic lignan extracted from sesame oil (*Sesamum indicum* L.) and sesame seeds (Siriwarin and Weerapreeyakul, 2016). Sesamol possesses antioxidant (Joshi et al., 2005), anti-inflammatory (Shahidani et al., 2022), neuroprotective (Ren et al., 2020), hepatoprotective (Hsu et al., 2006) and anti-cancer (Majdalawieh and Mansour, 2019) capabilities. Sesamol scavenges free radicals and inhibits DNA damage and lipid peroxidation (Kumar and Singh, 2015). Sesamol also down-regulates the production of TNF-α and IL-6 in the macrophages activated by LPS through inhibiting NF-κB/MAPK signaling pathway (Wu et al., 2015). Moreover, sesamol reduces oxidative stress and inhibits apoptosis and inflammation upon cerebral ischemia in rats (Gao et al., 2017). 

The present study investigated the impact of sesamol on memory dysfunction, TNF-α level and oxidative stress biomarkers in LPS-administered rats. 

## Materials and Methods

**Animals**

Male Wistar rats (200-250g) were housed in a colony room under controlled temperature, 12hr light:dark cycles, and they had free access to food and water. The Ethic Committee for Animal Experiments at Isfahan University of Medical Sciences approved the study (IR.MUI.MED.REC.1398.571). 

**Experimental design**

Animals were assigned to four experimental groups (n=9), including control, LPS, LPS+Sesamol10 and LPS+Sesamol50 groups. LPS (*Escherichia coli*) was injected (1 mg/kg) on days 15-19, two hours before behavioral assessment in the Morris Water Maze (MWM) (Ammari et al., 2018). Sesamol was injected (10 and 50 mg/kg) two weeks before LPS injection, and 30 min prior to LPS injection on days 15-19. After the behavioural experiment, animals were euthanized by CO2 and then decapitated. The cerebral cortex and hippocampus were immediately dissected out and homogenized with 10% NaCl for cytokine and oxidative assessments. 

**MWM**

The maze was a circular pool with a diameter of 150 cm which was filled with water (23±1°C). A circular platform (diameter 10 cm) was placed 2 cm below the surface of water at the midpoint of southeast quadrant. During acquisition training, animals were trained to find the platform within 60 sec (4 trials/day × 4 days) with 60 sec intersession intervals. In each trial, a rat was released at one of the four starting points to find the platform. The software NeuroVision (TajhizGostar Co.) calculated the escape latency for each animal. A probe trial was carried out on the 5^th^ day to evaluate memory retention for the location of platform. The platform was taken off, and animals had permission to swim in the maze for 60 sec. The time spent in the southeast quadrant was recorded (Azmand and Rajaei, 2021).

**Cytokine level**

The levels of TNF-α were determined by an ELISA kit (eBioscience Co., USA). Hippocampal and cortical homogenates were centrifugated at 3000 rpm for 5 min, and then, supernatant was collected to detect TNF-α. The level of TNF-α is presented as pg/ml.

**Oxidative stress biomarkers**

Thiobarbituric acid reactive substances (TBARS) and total thiol level were measured in the cortical and hippocampal homogenates as explained before (Rajaei et al., 2013). 

**Statistical analysis**

Two-way repeated measures ANOVA and one-way ANOVA followed by Tukey’s test was used to analyze data. Data are expressed as mean±S.E.M. A value of p<0.05 was considered significant.

## Results

**The impact of sesamol on spatial learning and memory**

**Figure 1:**
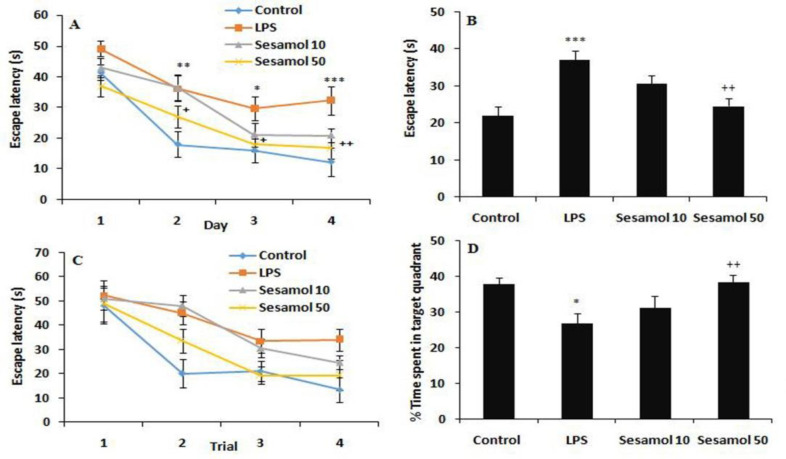
Effects of sesamol on the performance of spatial memory acquisition phase in Morris water maze, (A) Escape latency during 4 days, (B) overall escape latency, (C) escape latency on the first day, (D) performance in probe trial. Data are mean±SEM for nine animals in each group. *p<0.05, **p<0.01, and ***p<0.001 *vs *the control group and +p<0.05 and ++p<0.01 vs the LPS group.

Data analysis showed that the time to reach the hidden platform was reduced during four training days in experimental groups, demonstrating the acquisition of spatial learning (F(3,84)=41.21, p<0.001, Figure 1A). Moreover, the rats in the LPS group spent more time to reach the hidden platform in comparison with the controls (F(3,32)=13.02, p<0.001, Figures 1A and 1B), demonstrating a deficit in acquisition of spatial learning. Furthermore, treatment with sesamol (50 mg/kg) reduced the time during all training days in comparison with the LPS group (p<0.01, Figure 1A and 1B). These findings suggest that the LPS-mediated spatial learning and memory deficit was rescued by sesamol. Additionally, comparison of latencies on the first day did not show any significant change on the first trial in the experimental groups (Figure 1C). On probe test, the time spent in the southeast quadrant was reduced in the LPS group in comparison with the controls (F(3,32)=6.12, p<0.05, Figure 1D). Moreover, sesamol treatment (50 mg/kg) enhanced the time spent in the platform quadrant (p<0.01, Figure 1D) in comparison with the LPS group. 

**The impact of sesamol on TNF-α level**

**Figure 2:**
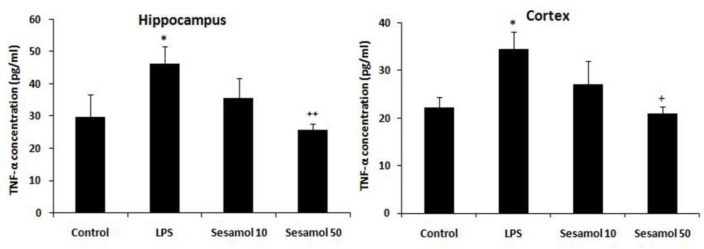
Effects of sesamol on TNF-α level in the hippocampus and cerebral cortex of experimental groups. Data are mean±SEM for nine animals in each group. *p<0.05 *vs* control group and +p<0.01 and ++p<0.01 *vs *the LPS group.

TNF-α level was enhanced in the hippocampus (p<0.05) and cerebral cortex (p<0.05) of the LPS group in comparison with control group (Figure 2). Moreover, sesamol (50 mg/kg) decreased the TNF-α level in the hippocampus (p<0.01) and cerebral cortex (p<0.05) in comparison with the LPS group (Figure 2). 

**The impact of sesamol on TBARS level**

**Figure 3:**
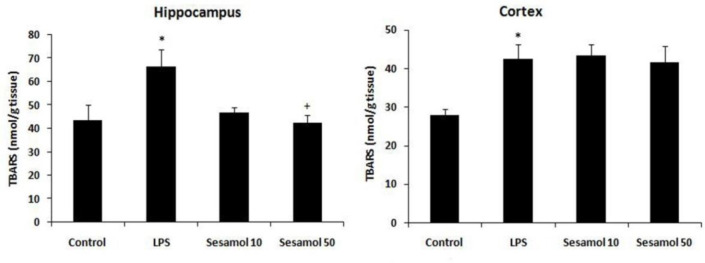
Effects of sesamol on TBARS level in the hippocampus and cerebral cortex of experimental groups. Data are mean±SEM for nine animals in each group. *p<0.05 *vs *the control group and +p<0.05 *vs *the LPS group.

The results demonstrated that the cortical (p<0.05) and hippocampal TBARS levels (p<0.05) were increased in the LPS group in comparison with the control group (Figure 3). Moreover, sesamol treatment (50 mg/kg) reduced the hippocampal TBARS levels in comparison with the LPS group (p<0.05, Figure 3). 

**The impact of sesamol on total thiol level**

**Figure 4:**
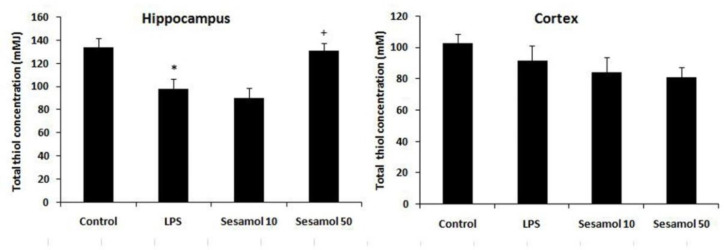
Effects of sesamol on total thiol concentration in the hippocampus and cerebral cortex of experimental groups. Data are mean±SEM for nine animals in each group. *p<0.05 *vs *the control group and +p<0.05 *vs *the LPS group.

Total thiol levels were decreased in the hippocampus of the LPS rats as compared to the controls (p<0.05, Figure 4). In addition, sesamol supplementation (50 mg/kg) enhanced the hippocampal total thiol level as compared to the LPS group (p<0.05, Figure 4). 

## Discussion

Our findings demonstrated that LPS alone induced brain inflammation, oxidative stress and deterioration of spatial learning and memory abilities. Moreover, supplementation of sesamol (50 mg/kg) ameliorated memory impairments by inhibition of brain inflammation and oxidative damage. 

Ample studies have indicated that brain inflammation is a critical factor for developing cognitive decline and neuronal damage in AD (Voet et al., 2019; Millington et al., 2014). Neuroinflammation could result in cognitive impairments due to nuclear retention of NF-κB and proinflammatory mediators release. Activation of glia and increased neuroinflammatory responses have been reported in patients with AD (Wyss-Coray, 2006). Experimental studies have also shown that neuroinflammatory responses induce cognitive impairments in rodents (Czerniawski and Guzowski, 2014). Systemic LPS injection causes neuronal damage in the hippocampus, and subsequently memory deficits (Valero et al., 2014; Batista et al., 2019). LPS induces strong microglia activation, up-regulates the expression of different proinflammatory cytokines such as TNF-α and IL-6, and eventually causes neuronal death (Monje et al., 2003). LPS identifies and binds to CD14/TLR4 complex, subsequently activates NF-κB and induces proinflammatory cytokines release (Park and Lee, 2013; Parajuli et al., 2012). In this study, subacute treatment with intraperitoneal injection of LPS for five days was used to develop an inflammation model. Our findings revealed that learning and memory performances were impaired in LPS-administered rats, evidenced by prolongation of the time spent to find the platform along with a decrement in time on probe trial. Comparison of latencies on the first day did not show any significant change on the first trial in experimental groups; however, it was enhanced on trials 2 to 4. This result indicates that LPS administration did not affect motor behaviour.

Our results also demonstrated that sesamol could enhance learning and memory as evident by decrement in escape latency and enhancement of time on probe test. In other words, sesamol treatment improved learning abilities and restored memory in LPS-administered animals. These effects indicated the protective action of sesamol against LPS-induced abnormalities. The memory-enhancing effects of sesamol have been shown in diabetic animals (Kuhad and Chopra, 2008) and streptozotocin-induced memory impairments (Sachdeva et al., 2015). 

Systemic LPS injection also increased brain TNF-α level, but the level of this inflammatory mediator was suppressed by sesamol. This result shows that sesamol possesses an anti-neuroinflammatory activity. This finding is in agreement with prior studies showing that sesamol inhibited the expression of inflammatory cytokines. For example, it was shown that sesamol prevented the production of TNF-α and nitrite in LPS-treated macrophages (Chu et al., 2010). Sesamol also reduced the mRNA expression of different proinflammatory factors such as TNF-α in cerebral ischemia (Gao et al., 2017). Therefore, sesamol exerts an anti-inflammatory action by inhibiting the release of TNF-α in the rat brain.

Considerable evidence indicates a strong association between oxidative stress, an imbalance between ROS production and elimination, and cognitive decline in AD (Campos et al., 2014; Barnham et al., 2004). The nervous system is susceptible to oxidative stress, since high levels of polyunsaturated fatty acids present in the brain make it more susceptible to lipid peroxidation and oxidative modification (Uttara et al., 2009). LPS can produce oxidative stress by releasing free radicals, which is considered a critical factor for memory decline following LPS administration (Ammari et al., 2018; Amraie et al., 2020). In accordance with this, our results indicated that LPS-induced memory deficits were followed by brain oxidative stress, as evident by enhanced levels of TBARS and decreased total thiol levels in the brain. In addition, supplementation of sesamol (50 mg/kg) for three weeks reduced the level of TBARS and enhanced total thiol level in the hippocampus, indicating the antioxidant activity of sesamol in the brain. Previous studies have also reported the neuroprotective action of sesamol by removing free radicals and decreasing lipid peroxidation in cerebral ischemia (Gao et al., 2017), diabetes (Kuhad and Chopra, 2008), and aluminium chloride and streptozotocin-induced cognitive impairments models (John et al., 2015; Sachdeva et al., 2015). It was also shown that treatment of aging mice with sesamol improves aging-related cognitive dysfunction by suppressing malondialdehyde production and enhancement of antioxidant enzymes in the hippocampus (Ren et al., 2020). Conclusively, the beneficial impact of sesamol on memory loss in this study could be partially attributed to the antioxidant activity of sesamol.

Evidence indicates that the cholinergic system plays an essential role in memory, and its dysfunction contributes to the pathology of neuroinflammation (Nizri et al., 2006). Degeneration of cholinergic neurons in the basal nucleus of Meynert occurs in early forms of AD, and is related to cognitive decline (Winkler et al., 1998). It was shown that LPS induces cholinergic neuronal loss (Houdek et al., 2014) and enhances acetylcholinesterase activity (Tyagi et al., 2010). *Sesame indicum* was shown to improve memory impairments induced by scopolamine in rats (Chidambaram et al., 2016). Additionally, the anti-cholinesterase activity of sesamol has been previously reported (Topal, 2019). Therefore, the advantageous effect of sesamol on memory function in LPS-injected rats may also be mediated via anti-cholinesterase activity and potentiating the cholinergic system. 

It should be considered that LPS administration can cause learning and memory impairments by multiple mechanisms, including inhibition of neuroplasticity (Adetuyi and Farombi, 2021), downregulation of BDNF mRNA (Ozdamar Unal et al., 2022), neurogenesis impairment (Cai et al., 2019), amyloidogenesis (Zhan et al., 2018), and apoptosis (Daroi et al., 2022). Evidence shows that sesamol stimulates neurogenesis (Bosebabu et al., 2020), enhances BDNF levels (Ren et al., 2018), inhibits the amyloidogenesis (Liu et al., 2017) and apoptosis (Gao et al., 2017). Thus, the above mechanisms may also be involved in the beneficial impact of sesamol on memory impairments following the LPS challenge.

Conclusively, supplementation of sesamol alleviated spatial learning and memory impairments in LPS-exposed rats. The neuroprotective influence of sesamol on LPS-induced memory impairments could be attributed to the inhibition of neuroinflammation and oxidative damage. Thus, sesamol may be used as a potent adjuvant in the treatment of memory impairments in AD due to its neuroprotective effects.

## Conflicts of interest

The authors have declared that there is no conflict of interest.
